# Prognosis of the Trauma Patients According to the Trauma and Injury Severity Score (TRISS); A Diagnostic Accuracy Study

**DOI:** 10.30476/BEAT.2020.84613

**Published:** 2020-07

**Authors:** Reza Hosseinpour, Amir Barghi, Saadat Mehrabi, Shirvan Salaminia, Paria Tobeh

**Affiliations:** 1 *Department of General Surgery, Clinical Research Development Unit of Beheshti Hospital, Yasuj University of Medical Sciences, Yasuj, Iran*; 2 *Clinical Research Development Unit of Beheshti Hospital, Yasuj University of Medical Sciences, Yasuj, Iran*; 3 *Department of Pediatrics, Yasuj University of Medical Sciences, Yasuj, Iran*

**Keywords:** Trauma Score Injury Severity Score (TRISS), Trauma, Prognosis, Mortality

## Abstract

**Objective:**

To investigate the prognosis and survival rates of a group of Iranian patients with traumatic injuries using the trauma and injury severity score (TRISS) model.

**Methods:**

In this prospective cohort study, all the patients with multi-trauma referring to the Yasuj Shahid Beheshti hospital during 2018 were included. The patients’ demographic information, trauma and history of previous illness were recorded. Vital symptoms including respiratory rate, heart rate, hypertension, pulse rate and Glasgow coma scale (GCS) score were assessed. The injury severity score (ISS) was calculated based on the type and location of the injuries and according to the abbreviated injury scale (AIS) classification. The survival probability of the patients was assessed according to the TRISS model.

**Results:**

Overall, 252 trauma patients were evaluated out of whom, 195 (77.4%) were men and 57 (22.6%) women. If we consider the TRISS score probability above 0.5 as the chance of being alive, the mortality rate was 6.75%, that was lower than our series (7.1%). The ISS score and GCS had a positive significant relationship with other variables except respiratory rate, body temperature and hospitalization. Revised trauma score (RTS) was significantly associated with other variables including age, GCS, hemoglobin, systolic blood pressure and respiratory rate. TRISS had an area under curve (AUC) of 0.988 indicating a high prognostic accuracy.

**Conclusion:**

The mortality rate was lower than that of being predicted by TRISS. This might be due to treatment effectiveness and care for traumatic patients leading to decreased mortality. TRISS had high prognostic accuracy in trauma patients. We also reported an association between hemoglobin and survival rate. Therefore, it seems that considering the laboratory parameters can be useful in patients with trauma.

## Introduction

Trauma is currently among the leading causes of mortality and morbidity worldwide being the first in developing countries [1, 2]. Socioeconomic development, changes in lifestyle and increasing life expectancy have led the changes of the diseases pattern and prognosis in these countries [3]. In fact, injuries are the most common causes of death among peoples 1-34 years of age that leading to disability and loss of life [4]. In developing countries such as Iran, road traffic accidents (RTA) are the most common cause of injury and mortality [5-7]. Defining the prognosis and survival of the patients with traumatic injuries is vital for management and development of trauma systems in order to improve the outcome and decrease the mortality and morbidity [8]. The variations between the outcomes of trauma in various centers is due to quality of care, different baseline characteristics and various severity of injuries. Thus, there should be a tool to compensate for baseline characteristics and trauma severity to enable us to determine the prognosis and outcome [8]. The trauma severity scores are defined for such purpose to quantify the amount of injury and to determine the outcome. 

Trauma and injury severity score (TRISS) is still considered the most commonly used toll for determining the outcome of patients with traumatic injuries [9]. TRISS has been introduced in 1983 for survival prediction of the patients with traumatic injuries which is weighted combination of age, injury severity score (ISS) and the revised trauma score (RTS) [10]. Several attempts have been made to improve the efficacy and readability of the TRISS model. These include careful consideration of the missing data [11], recalibration of the variables and co-variables [9] and also use of new or modified scores such as New Injury Severity Score (NISS) [12]. Taking all these together, the TRISS model is still the most commonly used tool for determining the outcome of trauma patients worldwide [13]. 

Trauma scoring system summarize the injuries severity in a single unit and provide a better classification of trauma patients in a common language to allow comparisons between hospitals and trauma centers [14]. Accident and injury recordings provide us with the right information to monitor and review the care system. This information is collected on the basis of certain criteria such as TRISS [11]. The TRISS model is used to evaluate the treatment and care of patients in which the survival rate is calculated based on their characteristics [10]. Therefore, recording the accident and injury provide us useful information for monitoring and reviewing the care system. Thus, the aim of this study was to investigate the prognosis and survival rates of a group of Iranian patients with traumatic injuries using the TRISS model.

## Materials and Methods

*Study population*

This prospective cohort study was conducted during a 6-month period from January to July 2018, in Shahid Beheshti hospital, a level I trauma center affiliated with Yasuj university if medical sciences. We included all the patients with multiple-trauma who presented to the emergency department (ED) of our center during the study period. We included either road or non-road traffic accidents. All the included patients were adults (≥18 years of age) and were transferred to the ED by EMS or by themselves. Those with burn injuries, suffocations, drowning and those who were referred to other centers for further evaluation, were excluded from the study. Those with unknown mechanism of injury, comorbidities and those who were dead on arrival were also excluded from the study. The study protocol was approved by the Yasuj University of Medical Sciences institutional review board (IRB) and medical ethics committee (IR.YUMS.REC.1396.193). All the patients or their legal guardians provided their informed written consents before inclusion in the study. 

*Study protocol*

All the patients were initially evaluated by a general surgery resident and the baseline characteristics were recorded into a standard data gathering form. We have recorded the demographic information, previous illness history and characteristics of the recent trauma and injury. Vital signs including blood pressure, respiratory rate, heart rate and Glasgow Coma scale (GCS) score were assessed. The damage to each organ was also recorded. The ISS criteria were measured based on the type and location of injury and based on the AIS classification. The RTS was also assessed and accordingly the TRISS model was designed for each patient and the survival rate and prognosis was determined and recorded. The patients were followed until the final outcome was recorded. The efficacy and diagnostic accuracy of the TRISS model was then determined accordingly. 

*The TRISS Model*

To estimate the survival rate of patients with TRISS, RTS, ISS and age score, patients with 3 age groups (<15, 15-45, >45 years) were entered in a formula based on trauma type. The coefficient was equal for age group of <15 and 15-45 years but the probability of survival in penetrating trauma is also calculated based on blunt trauma for the age group of less than 15 years. Patients’ survival and death probability were predicted by using the TRISS model. The number of predicted deaths were calculated from the probability of death with all patients. The ISS, RTS and TRISS in patients was calculated and was then used to calculate the TRISS using the following formula [10]:

B=B0 + B1(RTS)+B2 (ISS)+ B3 (age)

*Patient and Public Involvement*

Trauma is the leading cause of mortality and morbidity in 15-44-year age group in Iran. Thus, defining the prognosis and prognostic factors will help us understand the leading causes of mortality and morbidity in trauma patients. This enable us to provide appropriate programs for improving the quality of care to decrease the attributable mortality and morbidity. The high disease burden of trauma in our population and society makes it mandatory to study the factors. Trauma patients were involved in the current study during recruitment and follow-up. The patients or their legal guardians were informed of the research aims and benefits and they all agreed to participate. The results of the study will be sent to the participants and will also be distributed through the local social media to affect the disease burden and the outcome measures. 

*Statistical analysis*

According to the previously reported rate of trauma in the area [5,6], with 80% power and alpha coefficient equal to 0.01 we assumed that at least 220 patients are required to determine the diagnostic and prognostic value of the model. In order to compensate for non-evaluable patients, we include 252 patients. All the data was analyzed by statistical package for social sciences (SPSS Inc., Chicago, Illinois, USA) version 22.0. All the data are presented as mean ± SD and proportions as appropriate. The normality of the mean distribution was assessed by Kolmogorov-Smirnov test. The proportions were compared suing Chi-square test. The correlation of the parametric variables with normal distribution was assessed using Pearson’s correlation while for parametric without normal distribution Spearman’s was used. The correlation coefficient (r-value) was reported. The accuracy of the TRISS model for predicting the outcome was assessed using receiver-operating characteristics (ROC) curve (plot of sensitivity vs. 1⎼specificity) according to area under curve (AUC). AUC=1 indicates a perfect test, AUC>0.9 indicates high accuracy and AUC between 0.7 and 0.9 indicates moderate accuracy [15]. A 2-sided p-value of less than 0.05 was considered statistically significant. 

## Results

In this study, 252 trauma patients were included. Of these, 195 (77.4%) were men and 57 (22.6%) were women. Overall, 234 (92.9%) were blunt trauma and 18 (7.1%) were penetrating. When, 183 (72.6%) were discharged after examination, 51 (20.2%) were hospitalized and 18 (7.1%) died due to trauma and injuries. In most patients (89.3%) the GCS level was above 9. The mean TRISS score was 91.32 ± 24.00 and the max score was 99.7. The baseline characteristics of the patients is summarized in Table 1. 

The age group did not differ in mortality rates but TRISS index showed significant difference between age groups. Based on Spearman ranks correlation, there was no significant relationship between TRISS and mortality in the age group of 15-45 years but there was a significant relationship in other age groups (*p*=0.09 *vs.* *p*<0.001). ISS score had a significant negative relationship with other variables except respiratory rate, temperature and duration of hospitalization. ISS was negatively associated with survival (Table 2). Table 3 shows that RTS had significant correlation with variables except respiratory rate, temperature and duration of hospitalization. There was a significant negative correlation between ISS score, pulse rate and positive significance with other variables. TRISS score was significantly correlated with other variables except age, respiratory rate, temperature and duration of hospitalization. There was a negative significant correlation between TRISS and pulse rate and positive significance with other variables (Table 4). Mean age, systolic blood pressure, diastolic blood pressure and temperature was different in the two types of trauma. The mean of these variables was higher in blunt trauma (Table 5). 

**Fig. 1:**
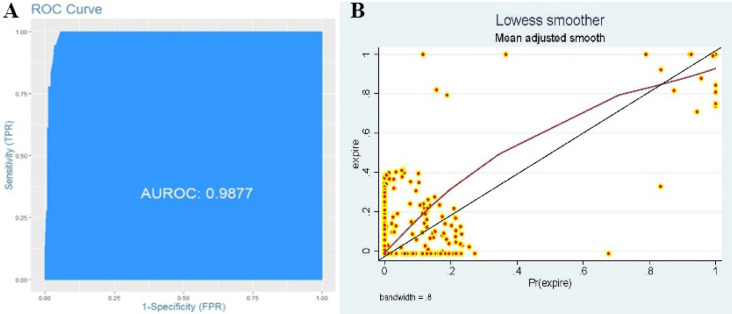
The receiver-operative characteristics (ROC) curve for determination of the trauma prognosis and survival according to the TRISS model. The area under curve (AUC) was highest in cut-off value of 0.40 (AUC=0.987) (A); the reliability curve demonstrating appropriate sharpness for TRISS model (B).

**Fig. 2:**
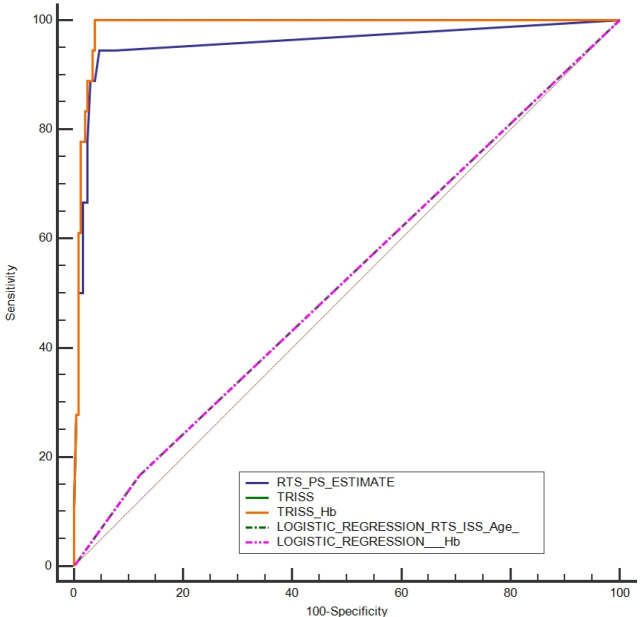
The receiver-operative characteristics (ROC) curve for determination of the trauma prognosis and survival according to the TRISS, TRISS +Hb and RTS models. As demonstrated TRISS and TRISS + Hb models have comparable area under curve (AUC).

From 234 patients who remained alive, TRISS model predicted 231 which determines the sensitivity of 98.7%. The mortality was predicted in 14 patients which calculated the specificity as 77.7%. We have also performed a confusion matrix with combing the TRISS model with different variables to improve the diagnostic accuracy (Table 6). As demonstrated adding the Hb adjusted for gender to the TRISS model decreased sensitivity and increased significantly the specificity. RTS has lower diagnostic accuracy compared to TRISS (*p*<0.001). However, the difference between the TRISS model and TRISS + Hb model was not statistically significant (*p*=0.788). The ROC curve analysis demonstrated that the best cut-off value for TRISS model was 0.36 in which the model reached its highest diagnostic accuracy (AUC=0.988). Both TRISS and RTS had appropriate diagnostic accuracy according to the AUC (0.988 *vs.* 0.957; *p*=0.273). The reliability curve also demonstrated appropriated sharpness and adding Hb did not affect the sharpness significantly (Figure 1). The ROC curve of the TRISS, TRISS + Hb and RTS models has also been demonstrated in Figure 2. 

## Discussion

This prospective cohort study focused on determining the outcome of the trauma patients admitted to a single Iranian center according to TRISS model. We also tried to investigate the diagnostic accuracy and reliability of the TRISS, TRISS + Hb and RTS models. We found that the TRISS and TRISS +Hb had the highest diagnostic accuracy according to the ROC curve and AUC. RTS had lower diagnostic accuracy while this difference was not statistically significantly. Overall, the TRISS model is considered a reliable and accurate model for determining the prognosis and survival rate of patients with multiple trauma.

In the current study, the most common causes of trauma mortality was road traffic accidents as demonstrated previously by several studies [5-7]. Recently, Yadollahi [16] demonstrated that the most common cause of trauma and the most common cause of death from trauma was traffic accidents. It was also found that an increase in the ISS index increases the risk of death in trauma patients, but the increase in GCS, revised trauma score (RTS) and TRISS indices reduces the risk of death in trauma patients. The TRISS indicator is better predictor of traumatic death than other indicators [16]. These findings are in line with ours. 

The findings reveal that road traffic accidents as the leading cause of trauma and falls from heights held the second rank, which is consistent with results from other studies [13, 17]. The most common site of injury was the head-and-neck region in our study, and the extremities had the highest frequency following that, which is justifiable considering the high prevalence of motor-vehicle accidents and falls from height. Some studies showed different results in this relation, which could be as a result of social and cultural differences in other communities [18, 19]. Approximately 46% of the deceased patients had been hospitalized for longer than 48 h; previous studies have presented consistent reports in this regard [18]. In regard to ISS, RTS and TRISS, our results showed that all these indices had a significant effect on risk of death due to trauma. Yadollahi [16] demonstrated that the risk of death increases by 10% for each unit increase in ISS, and for each unit increase in the indices of GCS, RTS and TRISS, risk of death would decrease by 40%, 80% and 10%, respectively. The present study reports a mean ISS of 13.43 ± 0.75, which is comparable to scores provided by other studies [20, 21]; this could be resulted by the fact that ISS is not determined in the cases of pre-hospital fatality. Furthermore, a regression analysis conducted by Ay *et al*., [22] revealed that ISS had significant effects on the mortality of trauma patients; several other studies, as well, have significantly determined increased.

The present study showed that ISS score and GCS had a positive significant relationship with other variables except respiratory rate, body temperature and duration of hospitalization. There was a negative significant relationship with ISS score and pulse rate and positive significant with other variables. RTS had significant relationship with other variables except pulse rate, body temperature and duration of hospitalization. In this regard, Champion *et al*., [23] showed that the mortality and morbidity rates significantly increased with increasing of TRISS. The relationship between ISS and mortality in traumatic patients has been studied by several studies [24, 25] that indicate the relationship between injury severity and mortality in trauma patients which is similar to the findings of the present study.

The present study showed that TRISS score which is an indicator to determine the probability of patient's survival, had a mean of 24±91.32 and the maximum score was 99.7. Therefore, we can say that TRISS is an effective treatment model and care for traumatic patients to reduce mortality. Yadollahi *et al*., [16] showed that increasing the ISS score will increase the risk of death in trauma patients but increasing GCS, RTS and TRISS will reduces the risk of mortality in trauma patients. The study found that TRISS is an indicator to predict the traumatic mortality. Based on the findings of the present study, TRISS score was significantly correlated with other variables except for age, respiratory rate, temperature and duration of hospitalization. Mean age, systolic blood pressure, diastolic blood pressure and body temperature were different in the two types of trauma. 

Results from a study by Schluter *et al*., [21] showed an ISS of lower than 14 in 3.5% of the fatality cases, and a GCS of between 3 and 4 in about 46% of them; both indices had an impact on risk of death. Overall, ISS, GCS, RTS and TRISS all can be used independently as predictors of mortality. These indices, used together or along with other triage indices, can become a more powerful, useful tool to estimate risk of death in trauma patients. As previously witnessed, results from our logistic regression analysis indicated the influence of TRISS on trauma-induced mortality; the analysis showed that TRISS can be an appropriate predictor of mortality in combination with increased heart rate and high blood pressure.

In the present study, there was a significant relationship with the two main determinants of TRISS which are ISS and RTS. Although in a study by Norouzi *et al*., [26] the relationship between RTS and survival rate was not significant. In this study TRISS calculated the survival probability for each patient. According to this calculation, 91.5% of patients were survived. Another study [27] showed that TRISS has an accuracy to predict mortality of trauma patients admitted to ICU and this model is more applicable because of easier calculation, trauma characteristics and quality of patient's independency care.

In the present study, there was a significant relationship between age and mortality rate that increasing of age will decrease survival rate. However, different result was found in a study of Kelly *et al*., [28]. Another study [29] also showed that increasing age has a significant effect on mortality which is similar to the findings of the present study. In the present study, gender had a significant relationship with Hb which the mean was higher in men that is physiological. Also, the study showed that there was no different between the age and accident group but the mortality probability based on TRISS was different in the groups of trauma that there was no significant relationship between predicted and observed deaths in the age group of 15 to 45 years.

We note some limitations to the current study which should be addressed in future studies. First, the sample size was approximately low which might have led to the type B error. The power analysis revealed an 80% power for the diagnostic accuracy analysis and AUC calculation. However, larger sample size populations could lead to improved quality of analysis and understanding the model. The other limitation was the fact the other models were not calculated and compared to the TRISS in the current study. While we aimed to evaluate this single model for prognosis of the trauma patients, comparison with other method could help us better understand the nature of the trauma prognosis. Overall, this is among the only available studies on the issue and could be referred for future studies. 

In conclusion, the present study indicates a better survival rate of patients. Therefore, it can be expected that the increase in the quality of medical services for trauma patients has led to the high observed of the TRISS index. It can be concluded that the care and treatment measures were effective and appropriate and it can be indicative of the treatment efficacy and care for traumatic patients to reduce mortality.

**Table 1:** Baseline characteristics of the 252 trauma patients included in the current study

**Variable **	**Value**
**Age (years)**	30.13 ± 18.2
**Transfer duration (minutes)**	35.35 ± 6.64
**White blood cell (WBC)**	11.85 ± 2.4
**Hemoglobin **	13.24 ± 2.33
**Systolic blood pressure (mmHg)**	113.6 ± 16.8
**Diastolic blood pressure (mmHg)**	72.62 ± 9.81
**Pulse rate **	88.82 ± 15.9
**Respiratory rate **	19.65 ± 4.01
**Temperature **	36.85 ± 0.32
**Glasgow Coma Scale (GCS)**	13.92 ± 3.10
**Injury Severity Score (ISS)**	13.43 ± 0.75
**Revised trauma score (RTS)**	7.7 ± 0.65
**Trauma Score Injury Severity Score (TRISS)**	91.32 ± 24

**Table 2:** Evaluation the Relationship between ISS Score and Variables in Pearson Test

**Variables**	**R**	**P-value**
**Age**	0.899	0.008
**Systolic Blood Pressure**	- 0.479	0.001
**Diastolic Blood Pressure**	- 0.461	0.001
**Pulse rate**	0.396	0.001
**Respiratory rate**	0.045	0.480
**Body Temperature**	- 0.039	0.545
**Duration of hospitalization**	- 0.061	0.339
**Glasgow Coma Scale (GCS)**	- 0.832	0.001
**Revised Trauma Score (RTS)**	- 0.822	0.001
**Hemoglobin (Hb)**	- 0.332	0.001
**Trauma and injury severity score (TRISS)**	- 0.931	0.001

**Table 3:** Evaluation the Relationship between revised trauma score (RTS) Score and Variables in Pearson Test

**Variables**	**R**	**P-value**
**Age**	0.141	0.025
**Systolic Blood Pressure**	0.572	0.001
**Diastolic Blood Pressure**	0.562	0.001
**Pulse rate**	- 0.463	0.001
**Respiratory rate**	0.047	0.455
**Body Temperature**	0.040	0.536
**Duration of hospitalization**	0.034	0.586
**Glasgow Come Scale (GCS)**	- 0.822	0.001
**Revised Trauma Score (RTS0**	0.963	0.001
**Hemoglobin (Hb)**	0.243	0.001
**Trauma and injury severity score (TRISS)**	0.918	0.001

**Table 4:** Evaluation the Relationship between TRISS Score and Variables in Pearson Test

**Variables**	**R**	**P-value**
**Age**	0.053	0.404
**Systolic Blood Pressure**	0.544	0.001
**Diastolic Blood Pressure**	0.536	0.001
**Pulse rate**	- 0.455	0.001
**Respiratory rate**	- 0.022	0.730
**Body Temperature**	0.046	0.477
**Duration of hospitalization**	0.033	0.601
**Injury Severity Score (ISS)**	-0.933	0.001
**Glasgow Come Scale (GCS)**	0.918	0.001
**Revised Trauma Score (RTS)**	0.225	0.001
**Hemoglobin (Hb)**	0.289	0.001

**Table 5:** Evaluation the mean of different variables in independent t-test according to type of trauma

	**Blunt (n=234)**	**Penetrating (n=18)**	**p-value **
**Age (years)**	30.82 ± 11.3	21.28 ± 9.21	0.032
**White blood cell (WBC)**	11.11 ± 2.3	10.48 ± 6.8	0.537
**Hemoglobin (Hb)**	13.20 ± 3.4	13.86 ± 5.6	0.244
**Systolic blood pressure (mmHg)**	114.23 ± 28.9	105.86 ± 39.3	0.035
**Diastolic blood pressure (mmHg)**	73.04 ± 15.9	67.22 ± 14.7	0.030
**Pulse rate (per min)**	88.78 ± 26.4	89.22 ± 31.4	0.910
**Respiratory rate (per min)**	19.67 ± 5.7	19.50 ± 7.4	0.866
**Body temperature (°C)**	36.87 ± 1.86	36.68 ± 2.57	**0.022**
**Injury severity score (ISS)**	13.23 ± 4.61	16.06 ± 5.21	0.517
**Revised trauma score (RTS)**	7.45 ± 2.23	7.16 ± 2.71	0.322
**Trauma and injury severity score (TRISS)**	91.57 ± 21.4	88.05 ± 18.6	0.550

**Table 6:** The results of confusion matrix with TRISS being calculated with hemoglobin (Hb) and gender adjusted Hb

**Items /models**	**TRISS **	**TRISS+ Hb**	**TRISS+ Hb adjusted for gender**	**RTS (PS)**
**Accuracy (%)**	97.22	96.43	97.22	94.05
**Confidence interval (%)**	94.36-98.9	93.33-98.35	94.36-98.9	90.37-96.63
**Sensitivity (%)**	98.72	98.29	98.29	99.15
**Specificity (%)**	77.78	77.22	83.33	27.75
**PPV (%)**	98.3	97.87	98.71	94.69
**NPV (%)**	82.35	76.47	78.95	71.43
**Prevalence (%)**	92.86	92.86	92.86	92.86
**Detection rate (%)**	91.67	91.27	91.27	92.06
**Detection prevalence (%) **	93.25	93.25	92.46	97.22
**Balanced accuracy (%)**	88.25	85.26	90.81	63.46
**kappa**	0.7851	.7237	0.7958	.0.375
**McNamara’s test **	1	1	1	0.009823
**AUC**	0.988	0.988	0.988	0.957
